# Adipose-derived stem cells improved mouse ovary function after chemotherapy-induced ovary failure

**DOI:** 10.1186/scrt231

**Published:** 2013-07-09

**Authors:** Min Sun, Shufang Wang, Yi Li, Ling Yu, Fang Gu, Changyong Wang, Yuanqing Yao

**Affiliations:** 1 Department of Obstetrics and Gynecology, Tangdu Hospital, Fourth Military Medical University, Xi’an, P.R. China; 2 Department of Blood Transfusion, The General Hospital of the People’s Liberation Army, Beijing, P.R. China; 3 Department of Obstetrics and Gynecology, First Affiliated Hospital of the General Hospital of the People’s Liberation Army, Beijing, P.R. China; 4 Department of Obstetrics and Gynecology, The General Hospital of the People’s Liberation Army, No. 28, Fuxing Road, Beijing 100875, P.R. China; 5 Department of Advanced Interdisciplinary Studies, Institute of Basic Medical Sciences and Tissue Engineering Research Center, Academy of Military Medical Sciences, Beijing, P.R. China

**Keywords:** POF, CTX, ADSC, Ovary, Stem cell therapy, Reproduction, ADSC transplantation

## Abstract

**Introduction:**

Young patients receiving chemotherapy occasionally face infertility and premature ovarian failure (POF). Numerous investigations reported that adipose-derived stem cells (ADSCs) transplantation could ameliorate the structure and function of injured tissues. The aim of this study was to explore the therapeutic efficacy of ADSC transplantation for chemotherapy-induced ovarian damage.

**Methods:**

Female mice were injected intraperitoneally with 50 mg/kg cyclophosphamide (CTX). After 15 consecutive days of injection, ADSCs were transplanted either directly into bilateral ovaries or via intravenous injection, and the ovaries were excised after either 1 week or 1 month of treatment. The follicles were counted and categorized, and ovarian histologic sections were stained for TUNEL. Ovarian function was evaluated by monitoring ovulation. ADSC tracking, microarray analyses, and real-time polymerase chain reaction (PCR) were used to assess the inner mechanism of injury and repair.

**Results:**

The ovarian function of mice exposed to CTX injection improved after ADSC transplantation. The population of follicles at different stages and ovulation significantly increased after the treatment. Immunofluorescence revealed reduced TUNEL staining. The tracking of ADSCs revealed that these cells did not directly differentiate into the follicle component. Microarray analyses indicated that changes in different groups of genes might affect follicle formation or ovulation.

**Conclusions:**

ADSC transplantation improved ovarian function. Our results suggest a potential mechanism for ADSC therapy.

## Introduction

Chemicals that are used to treat cancer are unquestionably beneficial as therapeutic agents. However, their side effect on the quality of life of female cancer survivors and their offspring cannot be ignored. Females are born with a finite number of undeveloped, primordial follicles. Chemicals that destroy primordial oocytes contained in the ovary can lead to premature ovarian failure (POF) or menopause [1]. POF is defined as secondary infertility with persistently elevated gonadotropin levels before the age of 40, with an estimated 1% incidence [2]. However, no clear definition exists for chemotherapy-induced ovarian failure, which is characterized by irreversible amenorrhea lasting more than 1 year after chemotherapy and follicle-stimulating hormone (FSH) levels more than 30 MIU/ml in the presence of a negative pregnancy test [3]. Apart from its direct effect on follicles and oocytes, chemotherapy has consequences, such as hot flashes, osteoporosis, cardiovascular and neurologic systems disease, sexual dysfunction, and the risk of infertility [4]. Cyclophosphamide (CTX), an alkylating agent, has irreversible cytotoxicity toward ovaries; in particular, it directly destroys oocytes and arouses follicular depletion [5,6].

Hormonal replacement therapy (HRT) has been used to treat common menopausal problems, but it increases the risk of cancer or recurrence in cancer survivors, forcing physicians to use alternative treatments. In recent years, interest has rapidly grown in the therapeutic potential of stem cells. Lee [7] concluded that bone marrow stem cells transplantation (BMT) rescued long-term fertility in CTX-treated female mice, and the oocytes of recipients are misidentified immune cells. Fu [8] demonstrated that BMT improves ovarian function in rats with chemotherapy-induced ovarian damage. However, bone marrow stem cells used in these previous studies were limited and caused trauma to the patient. The application of embryonic stem cells has ethical issues. ADSCs are a new type of MSC that is typically abundant in individuals, and their protective role in POF remains obscure.

Adipose-derived stem cells (ADSCs) are a type of multipotent adult stem cell isolated from adipose tissue. Cultured ADSCs can differentiate into multiple cell types, including osteoblasts, cartilage cells, adipocytes, myocytes, vascular endothelial cells, and neurons [9,10]. The biologic characteristics of ADSCs resemble those of adult stem cells from marrow [11]. Adipose tissue represents an abundant, poorly immunogenic, stably proliferative, low-injury and practical tissue source that holds great promise for autologous cell repair and regeneration. As a result, adipose tissue has become popular among researchers [12]. As the clinical applicability of ADSCs has been demonstrated in several clinical trials, including the treatment of ischemic myocardial dysfunction, kidney injury, carcinoma, and neuron system diseases, adipose tissue might provide an alternative source of stem cells for POF. In this study, POF was induced in mice by CTX treatment, and autologous ADSCs were directly injected into the ovaries or intravenously.

## Materials and methods

### Experimental animals

Eight-week-old female C57/BL6 mice and 8-week-old C57/BL6 female green fluorescent protein (GFP) transgenic mice were used in all of the experiments. The mice were purchased from Vital River Laboratories (Beijing, China). All of the animal procedures were conducted in accordance with the Animal Research: Reporting In Vivo Experiments (ARRIVE) guidelines for reporting animal studies. Ethical approval was obtained from the Ethics Committee of Military Medical Sciences (Beijing, China) for this study. The mice were fed a standard pellet diet and allowed free access to water. Vaginal smears were obtained daily. Only mice showing at least two consecutive normal 4– to 5-day vaginal estrus cycles were included in the experiments.

### Acquisition, identification, and labeling of ADSCs

ADSC isolation was performed according to the protocol previously reported [13]. In brief, subcutaneous adipose tissue was acquired from the inguinal region of mice. Next, adipose tissues were washed extensively with sterile phosphate-buffered saline (PBS) to remove contaminating debris and red blood cells. Next, the tissue was minced with scissors and digested with 0.1% type I collagenase (Sigma, US) in serum-free medium. The digestion was performed at 37°C for 30 to 60 minutes with gentle agitation. Then the enzymes were inactivated with an equal volume of α-MEM (Gibco, US) supplemented with 10% fetal bovine serum (FBS, Gibco), and the samples were filtered through a 200-μm mesh filter to remove debris. Then they were centrifuged at 600 *g* for 5 minutes to obtain cellular pellets. The cellular pellets were cultured in α-MEM/10% FBS, 50 U/ml penicillin, and 50 μg/ml streptomycin in a humidified incubator at 37°C with 5% carbon dioxide. Third-passage ADSCs were used for all of the transplantation experiments.

Surface-marker expression was analyzed with flow cytometry by using the following antibodies: fluorescein isothiocyanate (FITC)-conjugated mouse monoclonal antibodies against CD29, CD44, CD90, CD31, and CD45, and phycoerythrin (PE)-conjugated mouse monoclonal antibodies against CD34 (Biolegend, US). For flow cytometric analysis, adherent cells were detached by treatment with 0.25% trypsin-EDTA, neutralized with FBS-containing culture medium, and disaggregated into single cells by pipetting. The cells were incubated with mAbs for 30 minutes at 4°C, washed twice with PBS, resuspended in 0.5 ml PBS, and immediately analyzed by using an FACS Calibur flow cytometer (Becton Dickinson, US). A minimum of 2 × 10^5^ cells wase used for each sample, and cell Quest software was used for data analysis.

To confirm the multipotency of ADSCs, osteogenic and adipogenic differentiation were verified with Alizarin Red staining and Oil Red O staining, respectively [12]. In brief, to achieve osteogenic differentiation, cells were seeded onto the six-well plate at a density of 2 × 10^3^ cells/cm^2^. After 24 hours, the medium was replaced by osteogenic differentiation medium, and the cells were induced for 3 weeks. Then cells could be fixed and stained with Alizarin red; to achieve osteogenic differentiation, cells were seeded at a density of 1 × 10^4^/cm^2^. After reaching confluence, cells were incubated in adipogenic differentiation medium for 2 weeks. Then cells were fixed and stained with Oil Red O. The differentiation medium was changed every 3 days.

### Animal model establishment

To establish the POF model of chemotherapy-induced ovarian damage, adult female C57/BL6 mice were administered CTX (50 mg/kg) for 15 consecutive days via intraperitoneal injection.

### ADSC transplantation

After establishing the POF model, we randomly divided the mice into four equal groups. The WT group consisted of normal control mice that received no treatment. In the POF group, the mice were administered CTX. In the intravenous group, POF mice were injected intravenously with 1 × 10^6^ autologous ADSCs in a volume of 0.3 ml of 0.1 *M* PBS (pH 7.4). The injection was repeated again the following day. In the *in situ* group, 1 × 10^5^ ADSCs in 20 μl PBS were injected directly into the bilateral ovaries of POF mice with a microinjector.

### Ovarian follicle counts and morphologic analysis

ADSCs were administered either intravenously or *in situ*. The ovaries were collected 1 week and 1 month after treatment, and the follicles were detected and classified. Five representative sections from each ovary were selected. The ovaries were removed and fixed in 4% paraformaldehyde for at least 24 hours. After fixation, the ovaries were dehydrated, paraffin-embedded, serially sectioned at 5 μm, and mounted on glass microscope slides. Routine hematoxylin and eosin (H&E) staining was performed for histologic examination with light microscopy. Primordial, primary, secondary, and antral follicles were counted on every fifth section. Only follicles containing an oocyte were counted to avoid counting any follicle twice. Follicles were classified as follows: primordial follicle, oocyte surrounded by a single layer of squamous granulosa cells; primary follicle, intact enlarged oocyte with a visible nucleus and one layer of cuboidal granulosa cells; secondary follicle, two or three layers of cuboidal granulosa cells without antral space; early antral follicles, emerging antral spaces; and preovulatory follicles, which are largest of the follicular types and possess a defined cumulus granulosa cell layer [14].

### Mouse superovulation

Four groups of female C57/BL6 mice were superovulated 1 week or 1 month after transplantation via an intraperitoneal injection of 5 IU pregnant mare serum gonadotropin (PMSG), followed by intraperitoneal injection of 5 IU human chorionic gonadotropin (HCG) 48 hours later. The oocytes were collected from the ampulla portion of the oviduct 14 to 16 hours after HCG injection.

### TUNEL assay

An *in situ* Cell Death Detection Kit, POD (Roche, Germany), was used to detect apoptosis in the mouse ovaries, according to manufacturer’s instruction. Next, the nucleus was dyed with Hoechst 33324, and the sections were observed with fluorescence microscopy (Olympus, Japan). Apoptotic granulosa cells (GCs) in the ovary were stained green, and five random fields from each sample were counted (five samples/group).

### Cell-tracking studies

For cell-tracking studies, C57/BL6 female GFP transgenic mice were used as donors, and C57/BL6 female mice served as receptors. GFP-positive ADSCs were acquired as described previously [15]. The transplanted mice were killed 1 week and 1 month after transplantation of ADSCs. The ovaries were collected and made into frozen sections. The oocytes were collected from the ampulla portion of the oviduct 14 to 16 hours after HCG injection. The sections and oocytes were observed with fluorescence microscopy (Olympus).

### Microarray and real-time PCR analysis

RNA was isolated from mouse ovaries by using Trizol (Invitrogen) with standard methods. Labeling and hybridization were performed at the CapitalBio Company, according to protocols described in the 32 K mouse genome arrays user manual. The data were analyzed by using LuxScan 3.0 Image analysis software (CapitalBio Company, China).

Real-time quantitative PCR reactions were set up in triplicate by using the SYBR Green Real-time PCR Master Mix (Applied Biosystems, US) and run on a Bio-Rad CFX96 (US). The PCR primers were designed according to cDNA sequences in the NCBI database (Table 1). All of the gene-expression levels were normalized to the internal standard gene, *Gapdh*. For expression analysis, data from three replicates were analyzed by using the 2^−ΔΔCt^ method.

**Table 1:** Primers used in this study

**Gene**	**GB.accession**		**Primer sequence 5′ → 3′**	**Amplicon size (bp)**
*GAPDH*		F	GTTGTCTCCTGCGACTTCA	
		R	TGGTCCAGGGTTTCTTACTC	182
*Cxcr4*	NM_009911.3	F	TCAAGCAAGGATGTGACTTCG	
		R	GGCATAGAGGATGGGGTTCA	101
*Onecut2*	NM_194268.2	F	AGAGGGTTCTATGCCGGTCT	
		R	CTTTGCGTTTGCATGCTGCC	171
*Bpil3*	NM_199303.2	F	AGCACCCGAAGTCACTCTTC	
		R	AGTTAACTCCGCCTCGTGGA	161
*Angpt1*	NM_009640.3	F	TGCTAACAGGAGGTTGGTGG	
		R	GGCCCTTTGAAGTAGTGCCA	121
*Zcchc11*	NM_175472.3	F	CAGCCCAAACCCTTCTATGC	
		R	CGGATACCCTTGAGACAGCAG	143

### Statistical analyses

The statistical analyses were performed by using SPSS 14. The mean ± SEM of the data were calculated. Student *t* tests were used to determine the significance between two groups. One-way analysis of variance (ANOVA) with least significant difference (LSD) tests was used to determine significant differences between four groups. A *P* value < 0.05 was considered to be statistically significant.

## Results and discussion

### Cultivation and characterization of mouse ADSCs

**Figure 1:**
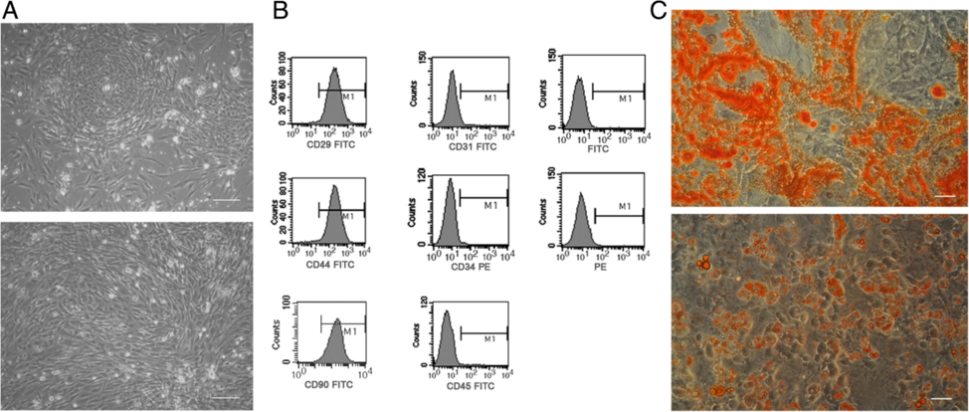
**The isolation and identification of ADSCs. (A)** The ADSCs exhibited typical fibroblastic morphology. **(B)** FCM analysis of ADSCs. The cells were positive for CD29, CD44, and CD90 and negative for CD31, CD34, and CD45. **(C)** ADSCs differentiate into osteoblasts and adipocytes. Scale bars: 100 μm.

Isolated cells were cultured in tissue-culture dishes. After 4 days in culture, the cells appeared to be spindle shaped and formed symmetric colonies (Figure 1A). After approximately three passages (Figure 1A), FACS analysis was used to identify the expression of surface markers. Most of the adherent cells expressed CD29, CD44, and CD90. Those markers were expressed in more than 95% of the population. In contrast, the majority of adherent cells were negative for CD31, CD34, and CD45 (Figure 1B). These ADSC were multipotent, as determined by their ability to differentiate into osteoblasts and adipocytes (Figure 1C).

Zuk [9] first reported the isolation of ADSCs from adipose tissue in 2001. As a novel source of MSCs, adipose tissue is typically abundant, poorly immunogenic, fast growing, minimally invasive, safe for autologous transplantation, avoids ethical problems, and is superior to other sources [13].

### Follicle number of the ADSC transplantation group increased after therapy

**Figure 2:**
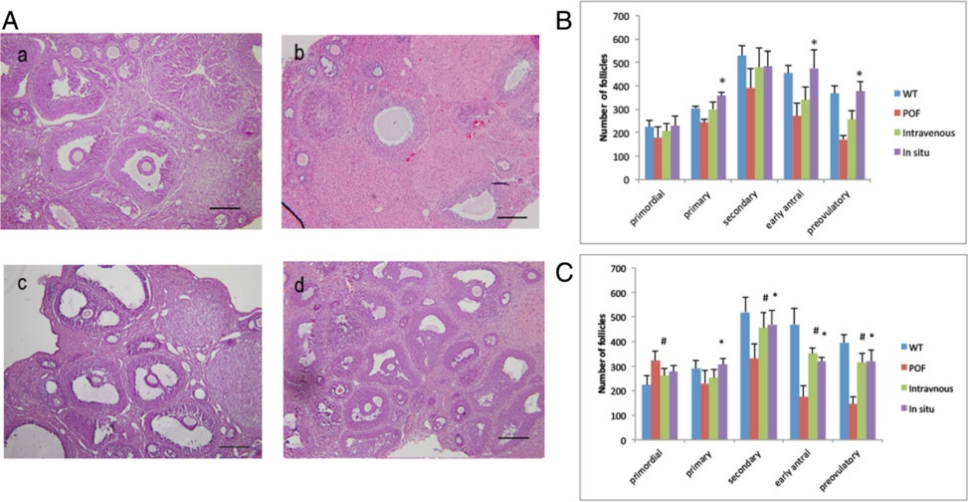
**The follicle number increased after transplantation. (A)** Hematoxylin and eosin staining. (**a**) Most of the follicles of WT and ADSCs were secondary and antral follicles. The population of follicles decreased significantly after injection with CTX (**b**) and recovered after the ADSCs both intravenous and *in situ* therapy (**c**, **d**). The POF model mouse had fewer follicles and most of them were primary and secondary follicles or had no function. **(B)** The number of follicles after one week. **(C)** The number of follicles after 1 month. Scale bars: 200 μm. (*P < 0.05 *in situ* versus POF group; ^#^*P* < 0.05 intravenous versus POF group).

Hematoxylin and eosin staining indicated that the POF group (Figure 2A(b)) had fewer follicles than the WT (Figure 2A(a)) and ADSC transplantation (Figure 2A(c, d)) groups. One week after transplantation, the numbers of follicles in the ovaries of female mice that received ADSCs via intravenous or *in situ* routes were increased. The ovaries of mice that received ADSC transplantation after chemotherapy possessed several hundred follicles at all stages of development. In contrast, the POF mice did not recover to the normal level within 1 month. Thus, both intravenous and *in situ* therapy significantly reduced ovary injury. After 1 week, the number of primary (357 ± 15.38), early antral follicles (476 ± 77.73) and antral follicles (377 ± 39.45) in the *in situ* group were significantly greater than those of the POF group (*P* < 0.05). Moreover, the amounts of other types of follicles in the intravenous and *in situ* groups were greater than those of the POF group, but these differences did not reach statistical significance. After 1 month, the number of primordial (278 ± 24.9), secondary (470 ± 57.45), early antral (320 ± 14.14), and preovulatory follicles (321 ± 42.49) in the *in situ* group, and the number of primary (252 ± 33.47), secondary (456 ± 62.29), early antral (352 ± 22.8), and preovulatory follicles (316 ± 38.47) in the intravenous group were significantly increased compared with the POF group (*P* < 0.05) (Figure 2C, D).

Several routes have been reported for the delivery of stem cells for ovarian repair, including intravenous [7] and *in situ* injection [8]. Both of these methods were used in this study and to compare their therapeutic effect. The results indicated that both methods improved ovary function and increased the numbers of follicles.

### Ovulation of the ADSC administration group increased after therapy

**Figure 3:**
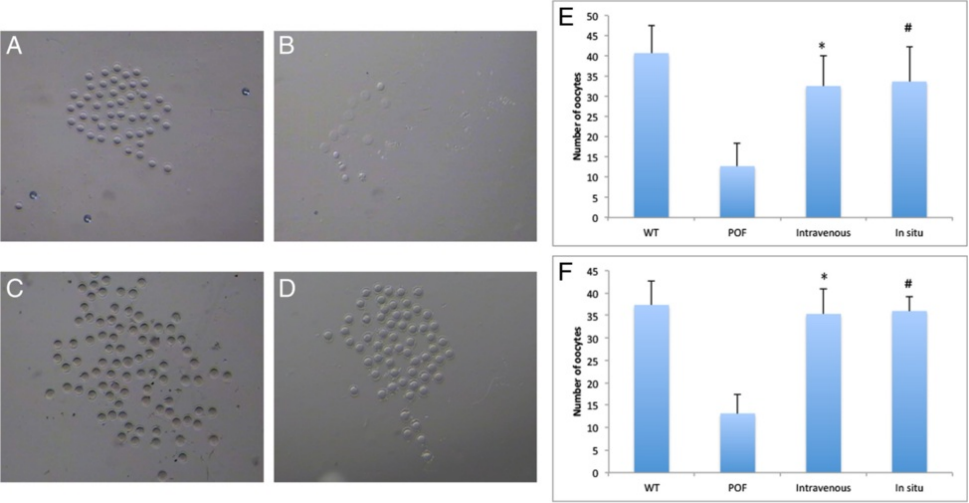
**The ovulation increased after therapy. (A)** The oocytes of the WT group. **(B)** The oocytes in the POF model exhibited abnormal morphology and number. **(C)** The oocytes of the intravenous group. **(D)** The oocytes of the *in situ* group. **(E**, **F)** The population of oocytes increased after 1 week and 1 month of ADSC therapy. (*P < 0.05 intravenous versus the POF group, ^#^*P* < 0.05 *in situ* versus the POF group).

Four female mice per group were injected with PMSG and hCG to induce superovulation. A subset of the oocytes in the POF group displayed an abnormal shape, with fragmentation and apoptosis in the cytoplasm. The ovulation number of the POF group (12.6 ± 5.6, *n* = 9) (Figure 3B) significantly decreased compared with the control group. After 4 weeks, ovulation remained low (13.2 ± 5, *n* = 6). However, in the intravenous (32.56 ± 7.53, *n* = 9, *P* < 0.05 versus POF group) and *in situ* groups (33.67 ± 8.63, *n* = 9, *P* < 0.05 versus POF group), ovulation increased 1 week after treatment (Figure 3).

### Apoptosis in the ovaries decreased after ADSC transplantation

**Figure 4:**
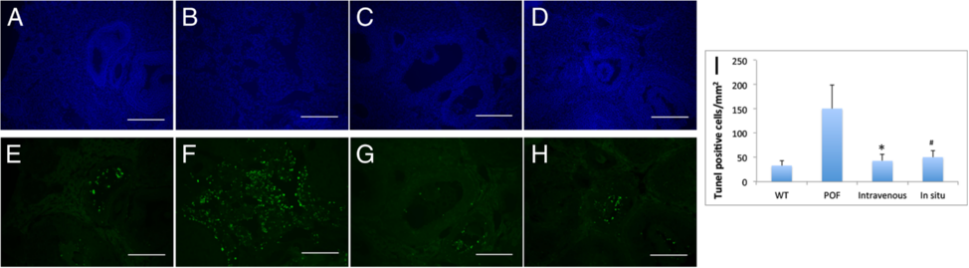
**The apoptosis of ovaries reduced after ADSC therapy.** The ovaries of WT group **(A**, **E)**, POF group **(B**, **F)**, the intravenous group **(C**, **G)**, and the *in situ* group **(D**, **H)**. The green stain indicates TUNEL-positive GCs. The blue stain indicates the nucleus. Data from one week demonstrate that apoptosis was attenuated by the transplantation of ADSCs. **(I)** The number of apoptotic GCs decreased after 1 week of ADSC therapy. Scale bars: 100 μm (*P < 0.05 intravenous versus POF group, ^#^*P* < 0.05 *in situ* versus POF group).

One week after ADSC transplantation, the number of cells that stained positive for TUNEL in ovary sections was quantified. The number of apoptotic GCs in the POF group (150.8 ± 48.4/mm^2^) was significantly higher than that of the other groups (*P* < 0.05). The number of TUNEL-positive cells in the intravenous (42.8 ± 13.7/mm^2^) and *in situ* (50.4 ± 13.1/mm^2^) groups was lower than that of the POF group (Figure 4). The apoptosis in the therapy group was reduced and closer to that of the control group (33.2 ± 10.3/mm^2^). Both of the therapeutic methods reduced host apoptosis.

Previous studies conclusively demonstrated that alkylating agents induce apoptosis in GCs and oocytes, and consequently, the loss of follicles [8,16]. This study indicates that both *in situ* and intravenous transplantation of ADSC rescues the apoptosis of GCs in ovary sections.

### ADSCs tracking *in vivo*

**Figure 5:**
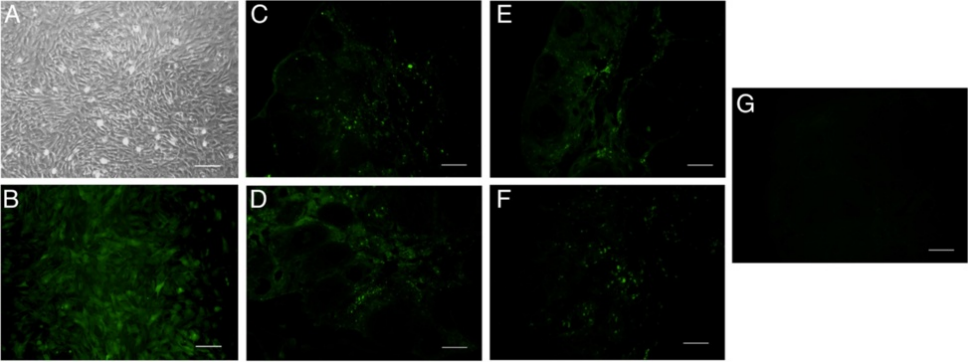
**ADSCs tracking *in vivo*. (A)** GFP (+)-ADSCs under a light microscope. **(B)** GFP (+)-ADSCs under a fluorescence microscope. **(C**, **E)** One week and 1 month after transplantation, ADSCs can be traced in the intravenous group. **(D**, **F)** One week and 1 month after transplantation, ADSCs can be traced in the *in situ* group. **(G)** ADSCs can not be traced in WT group. Scale bars: 100 μm.

**Figure 6:**
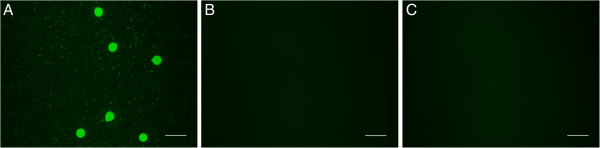
**Transplanted ADSCs were not formed into oocytes. (A)** Oocytes and cumulus cells from donor ovaries showed green fluorescence. **(B**, **C)** Oocytes and cumulus cells from recipient ovaries did not show green fluorescence. Scale bars: 100 μm.

Cell tracking is generally used to monitor stem cell homing and engraftment. One week and 1 month after transplantation, the injected GFP (+)-ADSCs were traced to the ovary, and GFP-positive cells were located in the interstitium but not in follicles (Figure 5). We also examined oocytes and cumulus cells from recipient ovaries, and we did not observe green fluorescence (Figure 6).

Careful observation is needed to determine whether stem cell transplantation constitutes regenerative cell therapy or cell-based cytokine therapy and to determine how long these injected cells can function in the ovary. Considering their strength and stability, GFP-positive ADSCs were used in this study. We found that ADSCs undergo a massive cell loss after intravenous injection, resulting in low signal intensity, which was consistent with previous reports. At 4 weeks, only a small portion of transplanted ADSCs could be detected in the intravenous group with immunofluorescence. We also observed that the interstitium in the therapy group exhibited fluorescent stem cells that had infused into the donor ovary, but these cells did not develop into follicles, including the GCs or the oocytes. This result indicates that transplanted ADSCs did not directly participate in follicle regeneration. Furthermore, a large number of engrafted cells died within 1 month after transplantation. Taken together, these results suggest that the direct regeneration of ADSCs played a minor role in the improved ovary structure and function.

### Microarray and real-time PCR analysis

**Figure 7:**
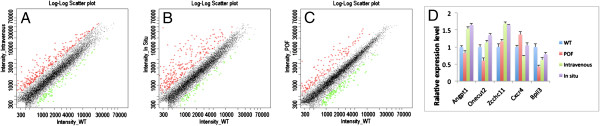
**Microarray and real-time PCR analysis. (A)** Gene expression in the intravenous group versus the WT group. **(B)** Gene expression in the intravenous group versus the WT group. **(C)** Gene expression in the *in situ* group versus the WT group. **(D)** Relative expression of genes. *Angpt1*, *Onecut2*, and *Zcchc11* were upregulated, and *Cxcr4* was downregulated; these results were in accordance with the microarray result.

The expression of 326 genes differed between the WT and the POF groups. One hundred sixty-six and 160 genes in the POF group were increased and decreased compared with the WT group, respectively. Compared with the intravenous group, 96 genes were upregulated, and 80 genes were downregulated in the POF group, whereas 150 genes were upregulated, and 75 genes were downregulated in the POF group compared with the *in situ* group (Figure 7) (accession number: GSE47741).

The pathways that were changed in the POF group and the ADSC transportation group compared with the WT group included genes related to the cytokine-cytokine receptor interaction, cell-adhesion molecules (CAMs), the transforming growth factor (TGF)-β signaling pathway, natural killer cell-mediated cytotoxicity, the MAPK pathway, and the Toll-like receptor signaling pathway. Some of those pathways are important for follicle and oocyte growth.

Some genes exhibited different expression levels between the ADSC therapy group and the POF group. *Zcchc11*, *Angpt 1*, *Onecut2*, among others, were upregulated after ADSC therapy in both therapy groups, whereas *Cxcr4* was downregulated, these results are in accordance with our microarray analysis. However, certain functions of these genes remain obscure (Figure 7).

ADSCs are known to secrete a number of cytokines, including high levels of angiogenic growth factors, such as HGF (hepatocyte growth factor), VEGF (vascular endothelial growth factor), PGF (placental growth factor), and TGF-β. ADSCs also exhibit moderate expression of FGF (fibroblast growth factor)-2 and Ang (angiopoietin)-1, as well as low levels of Ang-2 [17-20]. Some genes that regulate cytokines secretion changed. Furthermore, ADSCs improved ovarian function although they did not form into oocytes or GCs. Thus, the observed effect was likely due to cytokines produced by ADSCs. ADSCs may improve ovarian function via the action of paracrine cytokines.

Some cases of POF clearly have a genetic basis [21]. We used microarrays to evaluate genes related to ovarian function. The highly transcribed genes were related to functional groups such as stem cell cytokines, chemokine receptors, and transcription regulation.

The increased expression of *Angpt1* after therapy was in accordance with previous research. The change in Angpt1 and Angpt2 levels may be associated with follicular growth and angiogenesis during the preovulatory period [22]. Angpt1 is a cytokine secreted by stem cells. The secretion of Angpt1 makes ADSCs suitable for regenerative cell therapy [19].

CXCR4, an α-chemokine receptor specific for stromal-derived-factor-1 (SDF-1, also referred to as CXCL12), has potent chemotactic activity for lymphocytes and was downregulated after therapy. CXCR4/SDF1 may play an important role in retaining follicles in an inactivated state. The inactivation of CXCR4 may induce the growth of a primordial follicle into a mature follicle, and the CXCR4/SDF1 interaction can inhibit the primordial transition to a primary follicle in the neonatal mouse [23]. The downregulation of CXCR4 may promote the growth of primordial follicles into mature follicles.

The Zcchc11 enzyme is implicated in microRNA (miRNA) regulation. Zcchc11 is also important for postnatal survival and growth. Zcchc11 deficiency significantly decreased IGF-1 mRNA in the liver and insulin-like growth factor (IGF)-1 protein in the blood during the neonatal period [24]. IGF-1, a growth-hormone mediator, can stimulate GC proliferation, inhibit apoptosis, and promote follicular antrum formation [25,26]. IGF-1 may play a key role in sensitizing ovarian GCs to follicle-stimulating hormone (FSH) during terminal follicular growth [27] and also plays an endocrine and/or paracrine role in ovarian follicular development.

Apoptosis is likely a relevant mechanism in the genesis of POF in patients who have undergone chemotherapy or radiotherapy [28]. After ADSC therapy, the number of TUNEL-positive cells decreased significantly, and Zcchc11 expression was upregulated, indicating that ADSCs can reduce POF-mediated apoptosis.

ADSC therapy may be used in clinical applications for patients who have no distant metastasis. In this scenario, we can collect the patient’s stem cells and then reinfuse them after completing chemotherapy. For aggressive tumors, stem cells can be acquired after chemotherapy, although cell viability may decline, and the risk of cancer cell reintroduction may increase. More research is needed to evaluate the efficacy and safety of ADSC acquired after chemotherapy.

In regard to genetic defects, autologous ADSCs will presumably also carry the same genetic defect. Thus, ADSCs represent desirable target cells for gene therapy, as they are susceptible to transfection with exogenous genes. Experiments have confirmed that ADSCs can easily import exogenous genes *in vitro*, and these genes can be expressed efficiently and for long periods *in vivo*. Kucerova [29] used ADSCs as a drug cell carrier to successfully inhibit murine melanoma growth. Therefore, ADSCs are an important tool for the future of gene therapy.

## Conclusions

Our studies revealed that ADSCs significantly improved ovarian function after chemotherapy-induced ovarian injury. Our results suggest a potential mechanism for ADSC therapy. ADSCs could increase follicle number and oocyte number through gene-expression changes and the production of paracrine cytokines. ADSC treatment also decreased granulosa cells apoptosis. Therefore, ADSCs represent an alternative strategy for POF therapy and may be useful for future regenerative medicine and clinical applications.

## Abbreviations

ADSC: Adipose-derived stem cells; Ang: Angiopoietin; BMT: Bone marrow stem cells transplantation; CAMs: Cell-adhesion molecules; CTX: Cyclophosphamide; FBS: Fetal bovine serum; FGF: Fibroblast growth factor; FITC: Fluorescein isothiocyanate; FSH: Follicle-stimulating hormone; GC: Granulosa cell; GFP: Green fluorescent protein; HCG: Human chorionic gonadotropin; HGF: Hepatocyte growth factor; HRT: Hormonal replacement therapy; IGF: Insulin-like growth factor; miRNA: microRNA; PBS: Phosphate-buffered saline; PE: Phycoerythrin; PGF: Placental growth factor; PMSG: Pregnant mare serum gonadotropin; POF: Premature ovarian failure; SDF: Stromal-derived-factor; TGF: Transforming growth factor; VEGF: Vascular endothelial growth factor.

## Competing interests

The authors declare that they have no competing interests.

## Authors’ contributions

MS conceived of and performed all the experiments, analyzed the data, and drafted the manuscript. SW conceived of and performed animal experiments and revised the manuscript. YL was responsible for the molecular biology experiments, analyzed the data, and revised the manuscript. LY performed the cell experiments and analyzed the data. FG analyzed the data and drafted the manuscript. CW contributed essential reagents and tools and revised the manuscript. YY conceived of the research, contributed essential reagents or tools, and revised the manuscript. All of the authors read and approved the final manuscript.
